# Role of Circulating Exosomes in Cerebrovascular Diseases: A Comprehensive Review

**DOI:** 10.2174/1570159X21666230214112408

**Published:** 2023-05-18

**Authors:** Zhiwen Lu, Haishuang Tang, Sisi Li, Shijie Zhu, Siqi Li, Qinghai Huang

**Affiliations:** 1 Department of Neurovascular Centre, Changhai Hospital, Naval Medical University, Shanghai, 200433, China;; 2 Department of Nerurosurgery, Naval Medical Center of PLA, Navy Medical University, Shanghai, 200050, China;; 3 Department of Cerebrovascular Intervention, Shanghai General Hospital, Shanghai Jiao Tong University School of Medicine, Shanghai, 200080, China

**Keywords:** Exosome, cerebrovascular disease, biomarker, stroke, pathophysiology, therapy

## Abstract

Exosomes are lipid bilayer vesicles that contain multiple macromolecules secreted by the parent cells and play a vital role in intercellular communication. In recent years, the function of exosomes in cerebrovascular diseases (CVDs) has been intensively studied. Herein, we briefly review the current understanding of exosomes in CVDs. We discuss their role in the pathophysiology of the diseases and the value of the exosomes for clinical applications as biomarkers and potential therapies.

## 1 INTRODUCTION

Cerebrovascular disease (CVD) is a general term for a series of lesions in brain vessels, including arteries, capillaries, veins, and venous sinuses, the most prominent harm of which is stroke, one of the leading causes of death and disability worldwide [1]. According to the 2016 Global Burden of Disease Study, the global lifetime risk of stroke is approximately 25% from the age of 25 [2]. It is crucial to elucidate the pathogenesis of CVDs for their prevention and treatment, especially the underlying molecular mechanisms. A considerable number of studies have attempted to elaborate on the intrinsic mechanism from different perspectives. However, these studies seem to have focused on molecular changes within a specific cell type rather than information transfer between cells. Diseased cells may further affect adjacent cells and initiate a chain reaction that leads to an entire disease, which means that intercellular communication plays a vital role in this process.

Intercellular communication forms the basis for the coordination of biological processes in multicellular organisms. In addition to the function of widely accepted gap junction, chemical signaling, and contact signaling by plasma membrane-bound molecules in cellular communication, extracellular vesicles (EVs) have recently been recognized as novel players in cell-cell communication. Exosomes are a subgroup of EVs with a diameter of 40-160 nm [3], which differ from microvesicles in both size and biogenesis [4-6]. The generation of exosomes involves endosomes, internal vesicles, and multiple vesicle bodies. Finally, they fuse with the cell membrane and are secreted into the extracellular space. However, microvesicles form directly from the plasma membrane *via* outward budding [5, 6]. Although highly membrane-enriched tetraspanin family members, such as CD81, CD82, CD63, and CD9, are helpful in exosome identification, no specific markers have yet been identified [7]. According to Minimal Information for Studies of Extracellular Vesicles 2018 (MISEV2018), it is reasonable to describe EV subtypes by physical characteristics, biochemical composition, or cell origin instead of by exosomes unless reliable evidence of EV origin is provided [8]. However, the term exosome is often referred to as EVs in size range of 40-160 nm in the current literature, and we use the same definition in this review.

The initial understanding of exosomal functions came from the surface proteins. Surface proteins can either act as signal molecules to stimulate target cells [9, 10] or act as receptors to be transferred between cells [11, 12]. The biological function of exosomes depends more on a variety of bioactive molecules that they contain, such as nucleic acids, proteins, lipids, and metabolites. They are selectively encapsulated in exosomes during biogenesis [13]. Under normal physiological conditions, the components involved in exosome shuttling in different cells are relatively stable. However, the components vary significantly when stimulated by pathogenic factors, such as hypoxia [14], shear stress [15, 16], calcification [17], nicotine [18], and oxidized low-density lipoprotein(ox-LDL) [19]. Exosomes with abnormal contents are released into the blood, which may reflect the state of the diseases and become potential biomarkers. Exosomes not only indicate the type and physiological state of donor cells but also play a key role in the biological processes of recipient cells. Once transferred to recipient cells, they may cause pathological changes in the cerebrovascular and neurovascular units owing to their high permeability in the blood-brain barrier (BBB). However, this property also makes them a promising class of therapeutic agents for neurocyte protection and recovery.

Since the first report on exosomal nucleic acids [20], the studies on functional aspects of exosome has entered a new realm. From the translation of exosomal mRNA in recipient cells to the regulation of target genes through exosomal non-coding RNAs, the transfer of genetic messages provides a novel method for cell interaction. To date, a variety of exosomal nucleic acids, including mRNAs, non-coding RNAs, and DNAs, have been discovered [20-25]. Among these, exosome-derived non-coding RNAs, represented by miRNAs, lncRNAs and circRNAs, are the most extensively studied in CVDs [26-30].

MiRNAs are endogenous small RNAs with a length of approximately 17-24 nucleotides. They are important components of a complex gene regulatory network involved in cell proliferation, differentiation, and apoptosis [31]. MiRNAs, actively secreted by cells, are highly stable during circulation in multiple forms [20, 32-34]. Exosomal miRNAs are easy to be detected and extracted, and their value as diagnostic indicators of CVDs is rising [29, 30]. Apart from the role of biomarkers, miRNAs have also been shown to participate in the early protection and recovery of the nervous system after stroke [26]. LncRNAs are another group of non-coding RNA with more than 200 nucleotides and play key roles in biological functions through multiple levels of gene regulation, including epigenetic, transcriptional, and post-transcriptional regulation [35]. LncRNAs have been shown to be involved in the pathological processes of vascular disease by regulating vascular smooth muscle cell (VSMC) apoptosis [36] and inflammatory responses in atherosclerosis [28, 37]. CircRNAs are closed covalent loops lacking 3’ polyA tails and 5’ caps and are produced by the back-splicing of precursor mRNAs. CircRNAs regulate not only the transcription of parental genes [38] but the splicing of their linear cognates as well [39]. Meanwhile, circRNAs in the cytoplasm can work by binding to miRNAs [40], which is known as the ”miRNA sponge mechanism”. Interestingly, exosomal circ_ 0006896 likely regulates endothelial cells through this mechanism and promotes unstable atherosclerotic plaques [41]. In addition, circRNA can bind to proteins [42] and can also be translated into polypeptides [43].

This review provides an overview of CVDs-related exosomes with an emphasis on their potential as biomarkers and their effects on cerebral vessels and neurocytes in CVDs. This assists in revealing the biological mechanisms by which exosomes are involved in vascular and neuronal injury and protection. Additionally, we explored their potential for neurological recovery.

## 2 EXOSOMES AS BIOMARKERS OF CVDs

CVDs are insidious in their initial stages and are generally difficult to be diagnosed until neurological symptoms appear, when a stroke may have occurred. Angiographic monitoring contributes to early diagnosis and subsequent treatment; however, it is unsuitable for regular screening. Moreover, the assessment and prognosis of CVDs still depend primarily on symptoms and imaging examinations, and there is a lack of convenient and sensitive monitoring indicators. With an in-depth understanding, researchers have found that exosomes are highly stable in body fluids and can be easily extracted. More importantly, exosomes can reflect pathological changes in cells through abnormal cargo long before these changes are observed in organs. These characteristics are crucial for identifying ideal biomarkers. Recent studies have revealed the enormous potential of exosomes as biomarkers of CVDs (Table **1**).

### 2.1 Intracranial Aneurysms

Intracranial aneurysms (IAs), characterized by abnormal dilation of the arterial wall, carry a potential risk of bleeding known as aneurysmal subarachnoid hemorrhage (aSAH). Although it is difficult to observe the formation and growth of IA through imaging, changes in gene expression and exosomal non-coding RNAs during this process may be early warning signs. The miR-29a-3p and miR-145-5p are highly expressed in the plasma exosomes of patients with IA. Interestingly, miR-145-5p levels are significantly higher in ruptured aneurysms than in unruptured aneurysms, whereas those of miR-29a-3p are not, indicating different molecular mechanisms between IA growth and rupture [30]. Although clear evidence of aSAH can be revealed with computed tomography and lumbar puncture, false negatives owing to atypical features put patients at risk of rebleeding. Kalani *et al.* [44] performed LASSO analysis to generate a 24-miRNA classifier for discriminating SAH from parenchymal hemorrhage with high accuracy and specificity. These differentially expressed plasma exosomes may indicate a different etiology and are expected to help diagnose aSAH.

### 2.2 Moyamoya Disease

Moyamoya disease (MMD) is a CVD of unknown etiology, characterized by chronic progressive stenosis or occlusion of the terminal internal carotid artery, followed by the formation of an abnormal vascular network of the skull base. MMD is highly prevalent in East Asian countries and has attracted much attention because it is a critical cause of stroke in children and young adults [45]. However, early diagnosis of MMD, as well as the distinction of MMD from Moyamoya syndrome, remains challenging. Recently, the emergence of relevant exosomal miRNAs has shed new light on the diagnosis of MMD. To avoid interference from other systemic diseases, Wang *et al.* [46] extracted exosomes from cerebrospinal fluid samples for miRNA microarray analysis. RT-qPCR assay validation and ROC curve plotting suggested that the combination of miR-574-5p, miR-6165, and miR-6760-5p have superior diagnostic power.

### 2.3 Ischemic Stroke

Although there are still reports on exosomal non-coding RNA networks in other hemorrhagic CVDs [47], their role as biomarkers has been studied mainly in ischemic CVDs. Exosomes are sensitive to hypoxic-ischemic injury, which is reflected during minor strokes and even ischemic events on negative imaging [48, 49]. Xu *et al.* [50] showed that lnc-CRKL-2, lnc-NTRK3-4, RPS6KA2-AS1, and lnc-CALM1-7 are differentially expressed between patients with acute stroke and persistent NIHSS ≤ 4 and healthy controls. Recently, a novel algorithm based on the surface antigens of EVs was proposed to recognize transient ischemic attacks (TIA). Forty patients with suspected TIA symptoms were recruited as the training cohort, and 28 patients from another center were recruited for external validation. The patients were stratified according to the likelihood of an actual ischemic event based on the precise diagnostic score, and the EV-surface antigen profile was characterized in each group. The results indicated different EV-surface antigen profiles between patients who were adjudicated to have symptoms very likely caused by brain ischemia and those less likely to be a result of brain ischemia, indicating that they were helpful in identifying TIA [51].

For symptomatic ischemic stroke (IS), one of the major concerns is the judgment of the infarction phase, as the ischemic time window largely determines the treatment strategy. According to the current guidelines, intravenous thrombolysis is strictly limited to 4.5 h following symptom onset. However, this approach has not been fully utilized, especially in patients with wake-up strokes. Plasma exosomes may be helpful in assessing the duration of infarction. Wang *et al.* [52] investigated levels of plasma exosomal miRNA-21-5p and miRNA-30a-5p in IS patients in various phases, including the hyperacute, acute (including days 1-3 and 4-7), subacute, and recovery phases. According to their findings, miRNA-21-5p expression gradually increases from the acute phase to the recovery phase. MiRNA-30a-5p is significantly upregulated in the hyperacute phase (within 6 h) and decreases sharply during the process. Interestingly, compared with healthy controls, miRNA-21-5p levels also increase in the hyperacute phase and are higher than those in the acute phase. However, it may be necessary to observe dynamic changes in plasma exosomal RNA in each patient with IS to obtain more creditability. Compared to rigorous thrombolytic conditions, the indications for mechanical thrombectomy have expanded in recent years. As patients with small core infarction and large ischemic penumbra can still benefit from mechanical thrombectomy between 6-24 h after onset, a 24-h time window is recommended on the premise of accurate evaluation. A recent study showed that peripheral exosome circOGDH reflects the size of the ischemic penumbra after acute ischemic stroke (AIS), which facilitates rapid screening of patients who still require thrombectomy [53].

The etiology of IS is also closely related to subsequent treatment. According to the TOAST classification, IS is divided into five subtypes that are usually identified by imaging, blood tests, and previous medical history. Recently, plasma exosomes have been reported to indicate a specific subtype [54, 55]. Zhang *et al.* [56] compared patients with large artery atherosclerotic (LAA) stroke with healthy controls. All patients were matched to exclude interference from vascular risk factors, such as diabetes, hypertension, hyperlipidemia, and smoking. Eight lncRNA-related subnetworks were extracted in the discovery cohort (LAA: control = 5:5) by high-throughput sequencing, and 21 hub gene networks were selected according to GO and KEGG analyses. This was further verified in a validation cohort (LAA: control = 30:30). The results showed that exosomal lncRNA (Lnc_002015 and Lnc_001350)-related networks have clear advantages in identifying patients with LAA stroke. Another study, which included a larger validation cohort, revealed that four exosomal miRNAs (miR-369-3p, miR-493-3p, miR-379-5p, and miR-1296-5p) could also be used to identify LAA stroke. More interestingly, researchers noted that these miRNAs could even distinguish LAA stroke from atherosclerosis, which is considered the basis of LAA stroke [57]. Furthermore, serum exosomal miR-17-5p, miR-20b-5p, miR-27b-3p, and miR-93-5p expressions are significantly upregulated in patients with small vessel disease (SVD) compared to that in control participants. There are no differences in expression between other stroke subtypes and controls [58].

In addition, a growing number of studies have suggested that exosomes also indicate the severity and prognosis of stroke. Ji *et al.* [29] found that the levels of serum exosomal miR-9 and miR-124 are significantly higher in patients with AIS. Further studies showed that the levels of miR-9 and miR-124 are positively correlated with infarction volume and NIHSS score. Serum exosomal miR-134 is another indicator of IS severity. In addition to the positive correlation between NIHSS score and infarction volume, Zhou *et al.* [59] pointed out that a relatively high level of miR-134 is associated with poor outcomes. Moreover, elevated levels of miR-328 and miR-223 are associated with poor prognosis [60, 61]. Although these miRNAs have long been identified as potential biomarkers of IS, the predictive values of exosomal non-coding RNAs and free non-coding RNAs in the blood are different. Chen *et al.* [62] extracted serum miR-126 and exosomal miR-126 in rats with permanent and transient cerebral ischemia. The expression of serum miR-126 was found to be significantly higher in transient ischemic rats than in permanent ischemia rats 3 h after middle cerebral artery occlusion (MCAO). In this model, a surgical filament is inserted into the external carotid artery and threaded forward into the internal carotid artery until the tip blocks the origin of the middle cerebral artery (MCA), resulting in cessation of blood flow and subsequent cerebral infarction in the MCA territory, which can be used for permanent or transient occlusion. However, no detectable differences were observed in exosomal miR-126. Instead, the level of exosomal miR-126 is time-dependent in both transient and permanent ischemia rats, suggesting that it is more suitable as a biomarker of the phase of infarction than the severity of ischemia.

Prevention of ischemic events is an indispensable part of stroke management. Atherosclerosis may be more sensitive in identifying the occurrence and progression of future ischemic events. Previous studies have shown that the components of plasma exosomes are closely related to atherosclerosis pathology [63, 64]. This has also been observed in the cerebral vasculature. GLUT-1, LAT-1, and p-GP, selectively localized in cerebrovascular endothelial cells (EC), are significantly highly expressed in atherosclerotic cerebrovascular disease [65]. Jiang *et al.* [66] divided patients with intracranial atherosclerosis into two groups according to the recurrence of ischemic events under intensive medical management (IMM). After comparing plasma exosome components, researchers found a highly sensitive and specific exosomal miRNA expression profile in IMM non-responder patients. Additionally, the high expression of plasma exosomal miR-130a-3p in patients with asymptomatic carotid artery stenosis suggests the progression of stenosis [67].

## 3 ROLE OF EXOSOMES IN THE VASCULAR PATHOLOGY OF CVDs

In this section, we mainly discuss the exosomes involved in the pathological changes of the vascular wall in CVDs, rather than vascular development and abnormal arteriovenous anastomosis because the role of exosomes in the latter is poorly understood. ECs and VSMCs are not only the main components of the vascular wall but also the recipient cells of exosomes. They communicate *via* exosomes and receive them from macrophages, platelets, and neurocytes. However, various pathogenic factors gradually disrupt vascular homeostasis and communication, leading to endothelial dysfunction, inflammatory cell infiltration, and VSMC phenotypic transformation (Table **2**). VSMCs maintain the structural integrity and physiological function of the vascular wall with differentiated phenotypes in physiological states. However, they dedifferentiate under the stimulation of pathological factors. A dedifferentiated phenotype is a group of active VSMCs with diverse functions, including proliferation, inflammation, ossification, *etc*. [72]. More specifically, they present decreased expression of contract-related marker proteins, increased proliferation and migration, and increased synthesis and secretion of extracellular matrix, which are among the major pathological bases of proliferative vascular diseases, such as atherosclerosis, aneurysm, and restenosis after angioplasty.

### 3.1 Intracranial Aneurysm

The pathology of IA includes endothelial dysfunction, internal elastic lamina disruption, VSMC apoptosis, phenotypic transformation, extracellular matrix degradation, and chronic inflammation. Traditionally, macrophage infiltration is the primary cause of aneurysmal wall inflammation. However, this pattern was updated when macrophage-derived exosomes were found in abdominal aortic aneurysm (AAA) tissue in both humans and mice (the AAA mice model is constructed by alternately covering the infrarenal segment of the abdominal aorta with gauze soaked in CaCl_2_ and PBS). Moreover, the expression of matrix metalloproteinases 2 (MMP2) in cultured VSMCs increases under the influence of macrophage-derived exosomes. These *in vivo* and *in vitro* findings suggest that macrophage-derived exosomes are involved in the pathological process of aneurysms [73].

**Fig. (1):**
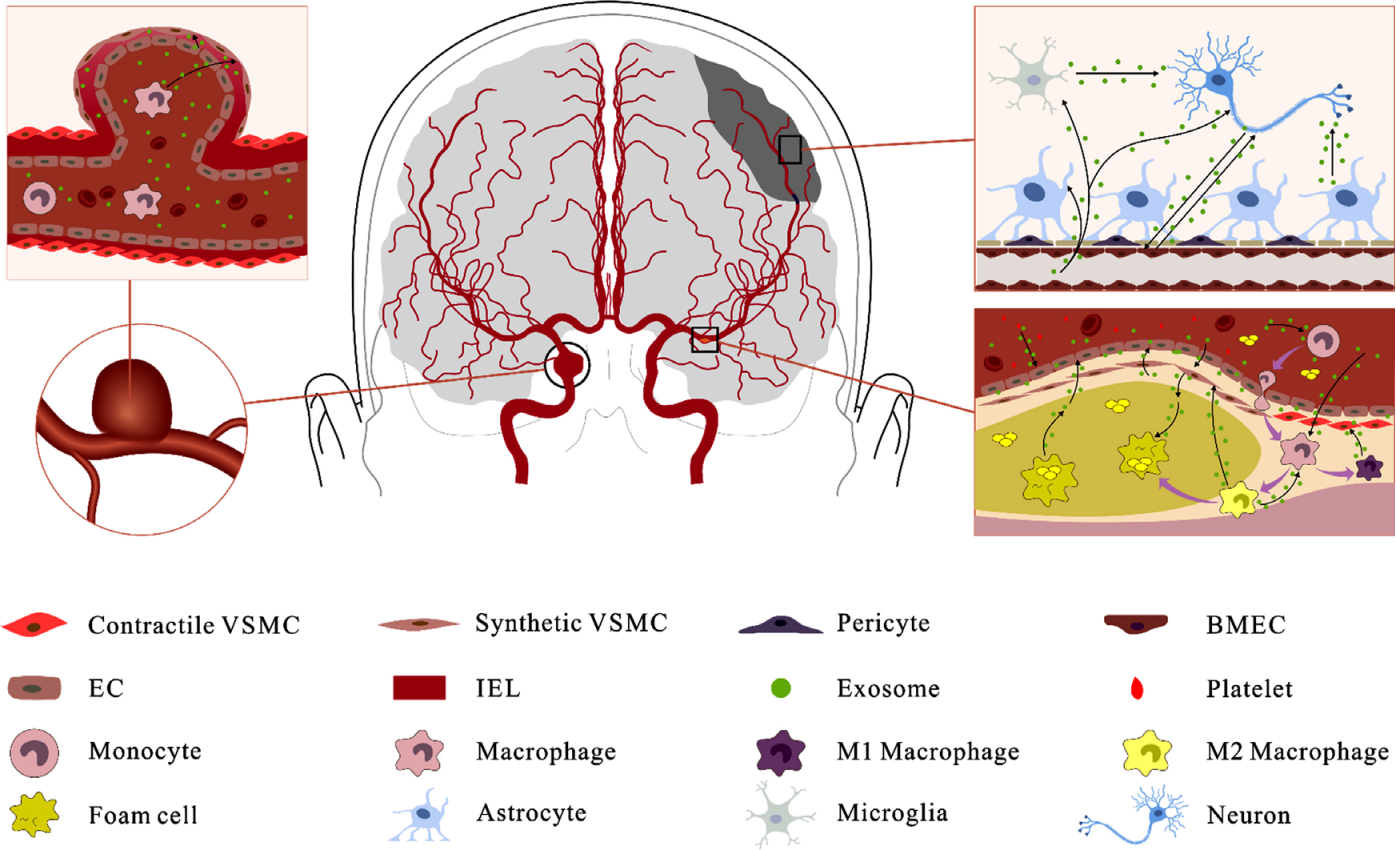
Exosomes play an important role in the pathogenesis of CVDs, such as atherosclerosis, intracranial aneurysms and ischemic stroke.

Exosome-mediated phenotypic transformation of VSMC promotes IA progression (Fig. **1**). Feng *et al.* [74] demonstrated that tumor-associated macrophages (TAMs) carrying miR-155-5p stimulate IA formation by promoting the proliferation and migration of VSMCs and the activation and infiltration of TAMs. However, additional evidence that exosomes regulate the VSMC phenotype comes from studies on other vascular diseases. EC-derived exosomes (EC-Exos) stimulate vascular cell adhesion molecule-1 (VCAM-1) expression and upregulate pro-inflammatory molecules in VSMCs, suggesting that exosomes may influence the VSMC phenotype [75]. Li *et al.* [76] provided direct evidence that downregulation of TET2 in EC-exos mediates phenotypic switching of VSMCs and intimal hyperplasia after arterial injury. Conversely, endothelial progenitor cell-derived exosomes (EPC-Exos) inhibit the synthesis phenotype of VSMCs [77].

Mesenchymal stem cells (MSCs) often inhibit the pathological process of IA and have been used in cell therapy for IA [78]. Interestingly, MSC-derived exosomes (MSC-Exos) inherit the therapeutic ability of their parent cells. IA treated with MSC-Exo miR-144-5p presents more ECs and fibrocytes and even increases the viability of ECs *in vitro*. This anti-IA effect is likely achieved by targeting phosphatase and tensin homolog (PTEN) proteins [79]. Anti-inflammation is another mechanism by which MSC-Exos functions. Exosomal miR-147 attenuates AAA formation by inhibiting macrophage activation [80]. Similarly, MSC-Exos could prevent IA rupture partly owing to a decrease in cytokine release, as well as tryptase and chymase activities of mast cells [81]. Moreover, bone marrow mesenchymal stem cell (BMSCs)-derived miR-23b-3p has been shown to inhibit IA formation by targeting Kruppel-like factor 5(KLF5) and suppressing nuclear factor κB (NF-κB). The contractile phenotype of VSMCs is also upregulated during this process, suggesting that inflammation is closely related to the phenotypic transformation of VSMCs [82].

### 3.2 Atherosclerosis

Atherosclerosis is characterized by atherosclerotic plaques in the inner lining of the large and medium arteries, resulting in vascular stenosis, tissue ischemia, and hypoxia. It is one of the most common causes of stroke globally and usually occurs in the carotid and intracranial arteries. The current understanding of atherogenesis involves EC injury, lipid deposition, inflammatory cell infiltration, foam cells, and fibrous plaque formation [83]. This process is inseparable from the communication between different cell populations. Several studies have revealed that exosomes play a crucial role in atherogenesis [63, 84] (Fig. **1**).

#### 3.2.1 Endothelial Cells

Atherosclerosis is initiated by low-density lipoprotein (LDL) deposition in the intima, followed by monocyte adhesion and activation. During this process, ECs lose their intrinsic anti-atherosclerotic ability and become dysfunctional owing to ox-LDL injury [85]. Inflammation is a pathological feature of EC dysfunction. Platelets are activated by EC injury and secrete exosomes that inhibit EC inflammation. MiRNAs are a group of active components in platelet-derived exosomes (PLT-Exos) that regulate EC inflammation. Yao *et al.* [19] suggested that miR-25-3p is abundant in PLT-Exos and inhibits ox-LDL-induced EC inflammation both *in vivo* and *in vitro*. This anti-inflammatory effect is closely related to the suppression of the NF-κB signaling pathway. Another study revealed that the level of miR-233 is elevated in thrombin-activated platelet exosomes, and PLT-Exo miR-233 reduces intercellular adhesion molecule-1(ICAM-1) expression in EC, suggesting that it inhibits EC inflammation [86].

EC dysfunction also manifests as a reduction in proliferation and migration, as well as the destruction of tight junctions. These factors may lead to increased endothelial permeability and enhanced atherosclerosis progression. Zheng *et al.* [87] revealed that VSMCs transfer KLF5-induced miR-155 to ECs *via* exosomes, thereby disrupting the integrity of tight junctions and endothelial barriers, which, in turn, promotes atherosclerosis progression. Moreover, transferred miR-155 in ECs was also shown to suppress EC proliferation/migration and re-endothelialization [87]. Fortunately, the protective effect of EPC-Exo on ECs provides therapeutic prospects for alleviating EC damage in atherosclerosis [85]. EPC-Exo miR-21-5p is a protective factor that rescues autophagic flux and promotes EC proliferation, migration, and tube formation. Ke *et al.* [88] asserted that EPC-Exo miR-21-5p protects against atherosclerosis by inhibiting SIPA1L2 expression in ox-LDL-induced ECs.

Although the angiogenic effect of exosomes contributes to neurological recovery after cerebral infarction and ECs repair in CVDs, it also increases plaque vulnerability, leading to sudden acute ischemic events [89, 90]. Bouchareychas *et al.* [91] indicated that angiogenesis is a cause of intraplaque hemorrhage. Li *et al.* [92] found that oxidative stress-activated EC exosomes promote angiogenesis, which is mediated by the downregulation of miR-92a-3p in recipient cells. Additionally, activated PLT-Exos were shown to promote angiogenic factors in ECs *via* miR-126 [93]. These findings suggest potential therapeutic targets to stabilize atherosclerotic plaques.

#### 3.2.2 Vascular Smooth Muscle Cells

VSMCs, the major contributors to plaque formation, participate in the entire process of atherogenesis. They decrease contractile protein expression and increase extracellular matrix (ECM) generation from the pre-atherosclerosis stage, leading to diffuse intimal thickening. With the further accumulation of ECM and LDL in the deep intima, extracellular lipid pools and foam cells are gradually formed until fibrous caps and the necrotic core consisting of lipid pools, dead VSMC, and macrophages appear [94]. During this process, macrophage foam cell-derived exosomes promote the phosphorylation of ERK and Akt in VSMCs in a time-dependent manner, leading to migration and adhesion of VSMCs in the intima [95]. Additionally, macrophage-derived exosomes promote atherosclerosis *via* smoking. Zhu *et al.* [18] revealed that in addition to the direct pro-atherosclerotic effects of nicotine on VSMCs, the retention of exosomes secreted by macrophages in the plaque might be another way to promote atherosclerosis. Further studies suggested that enriched miR-21-3p in retentive exosomes leads to the proliferation and migration of VSMC by targeting PTEN.

EC-Exos mediate the transfer of miRNAs to adjacent VSMCs and regulate their phenotype during atherosclerosis. EC-Exo miR-143 and miR-145 prevent VSMC dedifferentiation, whereas EC-Exo miR-92a stimulates atherosclerosis by promoting intimal hyperplasia [96, 97]. All of these processes are regulated by KLF2, a critical regulator of endothelial gene expression, which is a potential therapeutic target. Researchers have begun to explore the regulation of KLF2, and Lin *et al.* [96] have demonstrated that chrysin upregulates KLF2 expression and attenuates EC-Exo miR-92a.

#### 3.2.3 Macrophages

The recruitment and activation of monocytes and macrophages are essential for atherogenesis [98]. EC-Exo miR-10a inhibits monocyte activation by NF-κB suppression [99]. However, PLT-Exos may contribute to atherosclerosis by delivering pro-inflammatory cargoes to recruit monocytes [100].

Activated macrophages ingest cholesterol and release exosomes containing miR-146 to neighboring macrophages, which inhibits the migration of recipient cells and contributes to the pro-atherogenic phenotype of foam cells [101]. Foam cells are macrophages or VSMCs that phagocytose large amounts of ox-LDL. Their formation can be suppressed by ABCA1, a cholesterol outflow transporter that reverses cholesterol transport and reduces the lipid load [102]. Recently, a study showed that ABCA1 in macrophage foam cells is upregulated by perivascular adipose tissue-derived exosomal miR-382-5p [103]. These findings are promising for the prevention and treatment of atherosclerosis.

When activated, pre-foam macrophages exhibit strong phenotypic plasticity. They are generally classified into pro-inflammatory (M1-like macrophages) and anti-inflammatory (M2-like macrophages) phenotypes, which are determined by growth factors, cytokines, and specialized pro-resolving mediators [109]. Macrophages have the potential for dynamic phenotypic switching; in other words, they can undergo phenotypic transformation to alter their current functions under certain conditions [110]. Modulation of macrophage polarity in the arterial wall to the M2 phenotype has become another therapeutic strategy for atherosclerosis. Laura *et al.* [107] injected M2-polarized macrophage-derived exosomes into *Apoe^-/-^* mice and found that they promoted M2-like phenotypic transformation of macrophages in atherosclerotic lesions. Further research suggested that exosomal miR-99a-5p, miR-146B-5p, and miR-378A-3p contributed to M2-phenotype transformation, possibly by targeting tumor necrosis factor-alpha (TNF-α) and NF-κB in macrophages. Similarly, plasma exosomal lnc-MTGPTF-6:1 was demonstrated to regulate macrophage polarization. However, the knockdown of lnc-MTGPTF-6:1 was found to inhibit M1 polarization rather than promote M2 polarization [106].

### 3.3 In-stent Restenosis

Stent implantation has recently become a common strategy for the interventional treatment of IA and cerebral arteriosclerosis stenosis. Although stents effectively reduce IA recurrence and residual stenosis rate, in-stent restenosis has raised concerns.

The plasticity of VSMCs is a double-edged sword. Moderate repair after vascular injury promotes healing and restores normal physiological function. However, continuous excessive vascular remodeling is the pathological basis for restenosis after angioplasty. In-stent restenosis is closely related to uncontrolled proliferation and neointimal formation in VSMCs after vascular injury [111]. Macrophage-derived exosomes, including M1 and M2 macrophages, actively participate in vascular tissue repair following stent implantation. M2 macrophage-derived exosomes (M2-Exos) have been reported to promote VSMCs dedifferentiation and softening around stent struts. More importantly, M2-Exos stimulate the expression of c-KIT in VSMCs, a marker of stem cells [112]. Additionally, M1 macrophage-derived exosomes are involved in neointimal hyperplasia by transferring miR-222 to VSMCs to promote proliferation and migration [113].

## 4 ROLE OF EXOSOMES IN NEUROCYTE INJURY AND REPAIR IN CVDs

### 4.1 Ischemic Stroke

Sudden occlusion of the brain artery in IS results in an imbalance between glucose and oxygen supplements and disrupts the integrity of ECs and pericytes [114]. The inflammatory process, together with excitotoxicity and oxidative stress response, destroys the BBB, causing the dysfunction of neurons, astrocytes, and microglia [115, 116].

Due to the restricted treatment time window of clinical strategy and poor understanding of stroke-damaging mechanisms, not all patients can achieve favorable clinical outcomes in the long run. During stroke discovery, ischemic brain cells in the ischemic penumbra area, including neurons, astrocytes, microglia, and ECs, undergo partial functional recovery [117]. The border zone surrounding the core ischemic area triggers angiogenesis, neurogenesis, and oligodendrogenesis gradually [118-120]. Exosomes, as endogenous communicating and transporting systems, have great potential for the treatment of stroke. Different cells may generate different exosomes, which evoke different effects on various target cells [121]. The main advantages of exosome-based therapy in stroke treatment when compared to cell-based therapy include: 1) minor obstructive effects and immunogenicity, reduced minor thrombosis, and adverse immune reactions, 2) the ability to pass through the BBB, 3) high stability, which increases the possibility of large-scale production in therapeutic vesicle factories, and 4) the ability to be modified or engineered for different purposes [122-124]. The main components of exosome therapy for stroke are discussed in the following section.

#### 4.1.1 MSC-derived Exosomes

Emerging evidence has demonstrated that exosomes generated by stem cell-derived exosomes have therapeutic effects on neurological and functional recovery [125]. Huang *et al.* [126] suggested that MSC transplantation effectively improved functional recovery in MCAO rats. Among multiple cell types, MSC-Exos favor neurovascular remodeling during stroke recovery, most frequently in a paracrine manner [127]. Studies have demonstrated that intravenous administration of MSC-Exos after stroke can effectively enhance functional recovery and promote neurite remodeling, neurogenesis, and angiogenesis [128]. In addition to MSC-Exos, these exosomes can further stimulate neurons to release miRNAs, presumably promoting brain function recovery [129]. A previous study showed that MSC-Exo miR-455-5 reduced neuronal apoptosis and activated autophagy in a rat model [130]. Xin *et al.* [131] suggested that exosomes from MSCs mediate the transfer of miR-133b to astrocytes and neurons, which subsequently enhances neuronal remodeling and functional recovery after stroke. Dumbrava *et al.* reported that in aged rats, after occluding the distal MCA, MSC-derived small EVs promote neurological recovery and brain remodeling, and this effect may be mediated by increasing peri-infarct angiogenesis [132]. Wei *et al.* [133] reported that BMSC-derived exosomes improved post-stroke neuroplasticity in MCAO rats by promoting the proliferation and differentiation of neural stem cells. This mechanism may be related to the SOX10, Wnt/β-catenin, and endothelin-3/EDNRB pathways.

In addition to *in vivo* studies, the effect of MSC-induced exosomes on stroke was demonstrated in *in vitro* studies. Nalamolu *et al.* [134] revealed that human umbilical cord MSCs relieve ischemic damage in IS under oxygen-glucose deprivation (OGD) conditions. Xiao *et al.* [127] demonstrated that bone marrow-derived MSC-derived exosomes suppressed rat oligodendrocyte apoptosis *via* exosomal miR-134 by negatively regulating the caspase-8-dependent apoptosis pathway. Zhang *et al.* [135] showed that injection of human umbilical cord MSC-Exos attenuated microglia-mediated inflammation under OGD, whereas when miR-146a-5p was knocked down, this therapeutic effect was partially reversed. Their study also elucidated a potential cell-free therapeutic method for IS.

#### 4.1.2 Exosomes Derived from Other Cells

Many studies have confirmed the neuroprotective and neurorestorative roles of exosomes derived from other cell types, in addition to those of MSC-Exos in stroke treatment [136, 137] (Fig. **1**). Yang *et al.* [138] showed that exosomes from hypoxic pre-treated adipose-derived stem cells (ADSCs) attenuate stroke-induced brain injury, and the mechanism may involve the delivery of circ-Rps5 and promotion of M2 microglia/macrophage polarization. Similar effects of ADSC-derived exosomes have been reported in other studies [139, 140]. Exosomes from human urine-derived stem cells also enhance neurogenesis *via* the miR-26a-HDAC6 axis after ischemic stroke [141]. Zhong *et al.* [142] demonstrated that, under OGD manipulation, human umbilical vein endothelial cell (HUVEC)-derived exosomes protect neurons against ischemia-induced injuries, and the effects may be mediated by transferring miR-206 and miR-1-3p. Yu *et al.* [143] showed that exosomal miR-199a-5p derived from ECs attenuated apoptosis and inflammation in neural cells by inhibiting endoplasmic reticulum stress. Zang *et al.* [144] reported that microglia-derived exosomes promoted neuronal survival in an ischemic model. Another study showed that M2 microglia-derived exosomes attenuated ischemic brain injury and promoted neuronal survival *via* exosomal miR-124 [145]. Chen *et al.* [146] reported that ischemia-preconditioned astrocyte-derived exosomes decreased neuronal apoptosis and ameliorated neuronal damage by regulating the miR-7670-3p/SIRT1 axis. Similar effects of astrocyte-derived exosomes on neuronal apoptosis were confirmed in another study, and the specific mechanism was mediated by transferring miR-190b [147].

#### 4.1.3 Exosome Modification in Stroke Therapy

Exosomes with high stability, low immunogenicity, and high efficiency are ideal vehicles for transporting exogenous molecules and compounds to target cells. Modification of neuro-targeted exosomes may be an ideal therapeutic method for stroke treatment. Kim *et al.* [148] reported that an exosome linked to a receptor for an advanced glycation end-product binding peptide was developed as a hypoxia-specific carrier that could exert neuroprotective effects in the ischemic brain. Magnetic extracellular nanovesicles derived from iron oxide nanoparticle-harboring MSC effectively promote therapeutic outcomes in ischemic stroke [149]. Guo *et al.* [150] studied bioengineered neuron-targeting exosomes conjugated to a monoclonal antibody against GAP43 to promote the targeted delivery of quercetin to ischemic neurons with high GAP43 expression. The results showed that this method might serve as a promising therapeutic drug delivery system for reactive oxygen species scavenging. Curcumin is an anti-inflammatory molecule that can be encapsulated in exosomes. Engineered c(RGDyK)-conjugated exosomes target the lesion of the ischemic brain. Curcumin loaded onto c(RGDyK)-conjugated exosomes strongly suppresses the inflammatory response and apoptosis in ischemic lesions [123]. Zhang *et al.* [151] used the same c(RGDyK)-conjugated exosomes and found that the targeted delivery of miR-210 exerts angiogenic effects in cerebral ischemia in a mouse MCAO model. In addition to miRNA, siRNA can be delivered to stroke lesions by encapsulation in exosomes. Kim *et al.* [152] reported that high-mobility group box 1 (HMGB1) small interfering RNA (siRNA) delivered into ischemic brains by the intravenous administration of rabies virus glycoprotein peptide-decorated exosomes effectively reduced apoptosis levels in the ischemic brain (Table **3**).

### 4.2 Hemorrhagic Stroke

Subarachnoid hemorrhage (SAH) accounts for approximately 10% of acute stroke cases and is typically caused by IA rupture. Even with the rapid development of medical treatment and improvement of neurological intensive care, the mortality rate of SAH events caused by ruptured IAs is 45%, and approximately 30% of the remaining survivors still have serious disabilities [153, 154]. The damaging mechanisms of SAH can be mainly ascribed to early brain injury (EBI), early cerebral vasospasm, and delayed brain injury, which account for unfavorable clinical outcomes after SAH [155].

Several lines of evidence have demonstrated the relationship between exosomes and SAH. Chen *et al.* [26] revealed that the CX3CL1/CX3CR1 axis exerts protective effects after SAH by delivering exosomal miR-124 to microglia and attenuating microglial activation and neuroinflammation. Xiong *et al.* [156] reported that exosomes from BMSCs exert anti-inflammatory and anti-apoptotic effects through miRNA129-5p, which further alleviates EBI after SAH. Another study confirmed the anti-inflammatory and anti-apoptotic roles of MSC-Exos miRNA26b-5p in SAH *via* the MAPK/ STAT3 signaling pathway [157]. Systemic exosomal miR-193b-3p delivery also attenuated neuroinflammation in EBI after SAH in mice by acetylation of NF-κB p65 *via* suppressed expression and activity of HDAC3 [158].

## CONCLUSION

Exosomes widely participate in the physiological and pathological processes of CVDs. Increasing research on exosomes in CVDs has revealed their promising role as biomarkers and therapies. Modified exosomes further extend their functional roles. However, studies on therapeutic aspects, dose safety issues, and accurate time windows are required prior to application in clinical administration; thus, we should take a cautious attitude toward large-scale exosome production in therapeutic vesicle factories. In summary, exosomes have multiple advantages and show great potential for improving our understanding of CVDs mechanisms and treating CVDs; hence, they are worthy of further research.

## Figures and Tables

**Table 1:** Potential biomarkers of exosomes in CVDs.

**Markers**	**Disease**	**Role**	**References**
miR-152-3p	AIS	Related to the severity of infarction and the acute phase of stroke; predicts LAA stroke	[55]
miR-134	AIS	Related to the severity of infarction and prognosis	[59]
miR-21-5p, miR-30a-5p	AIS	Distinguishes the infarction phases	[52]
miR-9, miR-124	AIS	Related to the severity of infarction	[29]
miR-223	AIS	Related to the severity of infarction and short-term outcomes	[61]
miR-126	AIS	Distinguishes severe permanent ischemia from mild injury after transient ischemia	[62]
miR-422a, miR-125b-2-3p	AIS	Distinguishes the infarction phases	[68]
Combination of lnc-CRKL-2, lnc-NTRK3-4, RPS6KA2-AS1, and lnc-CALM1-7	AIS	Early detection of acute minor stroke	[50]
lnc_002015 and lnc_001350 related networks	IS	Diagnoses LAA stroke	[56]
a caspase-recruitment domain	IS	Reflects inflammatory response	[69]
miR-328-3p	IS	Predicts short-term prognosis	[60]
miR-17-5p, miR-20b-5p, miR-93-5p, miR-27b-3p	IS	Identifies the SVD subtype of ischemic stroke	[58]
miR-27b-3p	IS	Identifies subtype of stroke	[44]
Synaptopodin	Acute hypoxic-ischemic encephalopathy	-	[70]
miR-450b-5P	TIA	-	[48]
mo-miR-122-5p, mo-miR-300-3p	TIA	-	[49]
EV-surface antigen	TIA	An algorithm based on an EV-surface-antigen-specific signature that might aid in the recognition of TIA	[51]
miR-27b-3p, miR-199b-3p, miR-130a-3p, miR-221-3p, miR-24-3p	carotid stenosis	Predicts asymptomatic carotid stenosis progression	[67]
miRNAs (miR-122-5p, miR-192-5p, miR-27b-3p, miR-16-5p, miR-486-5p, miR-30c-5p, miR-10b-5p, miR-10a-5p, miR-101-3p, and miR-24-3p)	Intracranial atherosclerotic disease	Predicts the ineffectiveness of IMM	[66]
miR-630	SAH	-	[71]
miR-145-5p, miR-29a-3p	IA	-	[30]
miR-3679-5p, miR-6165, miR-6760-5p, miR-574-5p	MMD	-	[46]
lncRNAs and miRNAs	AVM	Identifies dysregulated exosomal lncRNA networks in AVM	[47]
EC-Exo and PLT-Exoscargo proteins	CVDs	-	[65]

**Abbreviations:** AIS, acute ischemic stroke; AVM, arteriovenous malformation; CVDs, cerebrovascular diseases; EC-Exo, endothelial cell-derived exosomes; EV, extracellular vesicle; IA, intracranial aneurysm; IMM, intensive medical management; IS, ischemic stroke; LAA, large-artery atherosclerosis; MMD, Moyamoya disease; PLT-Exos, platelet-derived exosomes; SAH, subarachnoid hemorrhage; SVD, small vascular disease; TIA, transient ischemic attack.

**Table 2:** Exosomes in the vascular pathology of CVDs.

**Origin**	**Cargo**	**Target**	**Recipient Cell**	**Disease**	**Effect**	**References**
MSC	miR-144-5p	PTEN	EC	IA	Increases viability of ECs	[79]
TAM	miR-155-5p	GREM-1	VSMC	IA	Promotes IA formation	[74]
MSC	NA	NA	mast cell	IA	Prevents the rupture of intracranial aneurysm	[81]
BMSC	miR-23b-3p	KLF5	VSMC/Th17	IA	Inhibits IA formation	[82]
Neuron	miR-132	eef2K	EC	Intracranial hemorrhage	Regulates the expression of VE-adhesion	[104]
NA	NA	NA	EC	MMD	Potential molecular mechanisms underlying the pathogenesis of MMD patients	[105]
Platelet	Chemokines, HMGB1	NA	EC/macrophage	AS	Contributes to thrombosis and atherosclerosis	[100]
Macrophage	miR-146a	IGF2BP1/HuR	macrophage	AS	Reduces macrophage migration to promote atherosclerosis	[101]
NA	lnc-MRGPRF-6:1	TLR4	macrophage	AS	Regulates macrophage polarization	[106]
EC	TET2	NA	VSMC	AS	Promotes neointimal formation, pro-proliferation, and migration	[76]
Macrophage foam cell	NA	ERK/AKt	VSMC	AS	Promotes VSMC migration and adhesion	[95]
Macrophage	miR-21-3p (nicotine-stimulated)	PTEN	VSMC	AS	Increases VSMC migration and proliferation	[18]
EC (oxidative stress-activated)	NA	miR-92a	EC	AS	Stimulates angiogenesis by inhibiting miR-92a-3p expression	[92]
EC	miR-92a	KLF2	EC	AS	Promotes intimal hyperplasia	[96]
VSMC	miR-155	Tight junction protein	EC	AS	Destroys tight junctions and the integrity of endothelial barriers; increases endothelial permeability	[87]
Platelet	miR-126	NA	EC	AS	Promotes the proliferation and migration of HUVECs	[93]
EC	miR-10a	NF-κB	macrophage	AS	Anti-inflammation	[99]
Perivascular adipose tissue	miR-382-5p	ABCA1, ABCG1	macrophage	AS	Reduces macrophage foam cell formation	[103]
Macrophage	miR-99a, miR-146b, miR-378a	NF-κB/TNF-α	macrophage	AS	Fosters M2 polarization; reduces hematopoiesis; and suppresses inflammation	[107]
EC	miR-143/145	NA	VSMC	AS	Regulates VSMC phenotype	[97]
EPC	miR-21-5p	SIPA1L2	EC	AS	Attenuates vascular endothelial injury; regulates lipid balance; and activates autophagy	[88]
EPC	NA	NA	EC	AS	Decreases atherosclerotic plaques and inflammatory factors; ameliorates endothelial dysfunction	[85]
Platelet	miR-223	ICAM-1	EC	AS	Inhibits ICAM-1 expression	[86]
Platelet	miR-25-3p (thrombin activated)	Adam10	EC	AS	Inhibits ox-LDL-induced EC inflammation and lipid deposition	[19]
EPC	NA	NA	EC	H/R injury	Inhibits EC apoptosis	[108]

**Abbreviations:** AS, atherosclerosis; BMSC, bone marrow mesenchymal stem cell; EC, endothelial cell; EPC, endothelial progenitor cell; HUVECs, human umbilical vein endothelial cells; H/R, hypoxia/reoxygenation; IA, intracranial aneurysm; MMD, Moyamoya disease; MSC, mesenchymal stem cell; NA, not applicable; SAH, subarachnoid hemorrhage; TAMS, tumor-associated macrophages; VSMC, vascular smooth muscle cells.

**Table 3:** Neuroprotective role of exosomes in IS and SAH.

**Origin**	**Cargo**	**Target**	**Disease**	**Effect**	**References**
MSC	NA	NA	IS	Promotes neurite remodeling, neurogenesis, and angiogenesis	[128]
MSC	miR-455-5	NA	IS	Reduces apoptosis of neurons and activated autophagy	[130]
MSC	miR-146a-5p	IRAK1/TRAF6	IS	Inhibits inflammation	[135]
ADSC	circ-Rps5	SIRT7/miR-124-3p	IS	Macrophage polarization	[138]
UDSC	miR-26a	HDAC	IS	Enhances neurogenesis	[141]
EC	miR-199a-5p	NA	IS	Attenuates apoptosis and inflammation	[143]
Microglia	miR-124	NA	IS	Promotes neuronal survival	[145]
Astrocyte	miR-7670-3p	SIRT1	IS	Reduces apoptosis of neurons	[27]
MSC	miRNA129-5p	NA	SAH	Anti-inflammation and anti-apoptosis	[156]
MSC	miRNA26b-5p	MAPK/STAT3	SAH	Anti-inflammation and anti-apoptosis	[157]

**Abbreviations:** ADSC, adipose-derived stem cell; EC, endothelial cell; IS, ischemic stroke; MSC, mesenchymal stem cell; NA, not applicable; SAH, subarachnoid hemorrhage; UDSC, urine-derived stem cell.

## LIST OF ABBREVIATIONS

AAA: Abdominal Aortic Aneurysm
ADSC: Adipose-derived Stem Cell
AIS: Acute Ischemic Stroke
AS: Atherosclerosis
aSAH: Aneurysmal Subarachnoid Hemorrhage
AVM: Arteriovenous Malformation
BBB: Blood-brain Barrier
BMSC: Bone Marrow Mesenchymal Stem Cell
CVD: Cerebrovascular Disease
EBI: Early Brain Injury
EC: Endothelial Cell
EC-Exo: Endothelial Cell-derived Exosome
ECM: Extracellular Matrix
EPC: Endothelial Progenitor Cell
EPC-Exo: Endothelial Progenitor Cell-derived Exosome
EV: Extracellular Vesicle
H/R: Hypoxia/Reoxygenation
HMGB1: High-mobility Group Box 1
HUVEC: Human Umbilical Vein Endothelial Cell
IA: Intracranial Aneurysm
ICAM-1: Intercellular Adhesion Molecule-1
IMM: Intensive Medical Management
IS: Ischemic Stroke
KLF: Kruppel-like Factor
LAA: Large Artery Atherosclerotic
LDL: Low-density Lipoprotein
M2-Exo: M2 Macrophage-derived Exosome
MCAO: Middle Cerebral Artery Occlusion
MMD: Moyamoya Disease
MMP2: Matrix Metalloproteinases 2
MSC: Mesenchymal Stem Cell
MSC-Exo: Mesenchymal Stem Cell-derived Exosome
NA: Not Applicable
NF-κB: Nuclear Factor Kappa B
OGD: Oxygen-glucose Deprivation
ox-LDL: Oxidized Low-density Lipoprotein
PLT-Exos: Platelet-derived Exosomes
PTEN: Phosphatase and Tensin Homolog
SAH: Subarachnoid Hemorrhage
SVD: Small Vessel Disease
TAMs: Tumor-associated Macrophages
TIA: Transient Ischemic Attacks
TNF-α: Tumor Necrosis factor-alpha
UDSC: Urine-derived Stem Cells
VCAM-1: Vascular Cell Adhesion Molecule-1
VSMC: Vascular smooth Muscle Cell
